# C-type natriuretic peptide chronic administration attenuates cardiac fibrosis and inflammation in spontaneously hypertensive rats

**DOI:** 10.1186/2050-6511-16-S1-A104

**Published:** 2015-09-02

**Authors:** E Santos Prentki, C Caniffi, G Bouchet, D Maglio González, J Toblli, MA Costa, C Arranz

**Affiliations:** 1 Physiology, School of Pharmacy and Biochemistry, IQUIMEFA-CONICET, University of Buenos Aires, Argentina; 2 Immunology, School of Pharmacy and Biochemistry, IDEHU-CONICET, University of Buenos Aires, Argentina; 3 Laboratory of Experimental Medicine, German Hospital, Buenos Aires, Argentina

## Background

Growing evidence shows that the expression and release of a number of inflammatory cytokines are increased in hypertension [1,2] and are also present at sites of cardiovascular fibrosis. In fact, many of these inflammatory mediators are responsible for the activation of collagen producing fibroblasts [3]. In addition, proinflammatory cytokines such as tumor necrosis factor-alpha (TNF-α) and interleukin-1 (IL-1) have shown to stimulate the release of C-type natriuretic peptide (CNP), indicating a potential role of CNP as a modulator of the inflammatory process [4]. The aim of the present study was to evaluate the effects of chronic CNP treatment on cardiac fibrosis and inflammation in spontaneously hypertensive rats (SHR).

## Experimental design and methods

12-week-old male SHR and Wistar rats were infused through osmotic pumps with CNP (0,75 µg/hr) or saline (S) for 14 days. Systolic blood pressure (SBP, mmHg) was recorded by tail-cuff method. At the end of the treatment, the left ventricle (LV) was extracted to determine left ventricular mass index (LVMI, g LV/mm tibia length), the myocyte area (µm^2^, Haematoxylin and Eosin-stain), fibrosis (% collagen, Picrosirius Red stain) and inflammation parameters (IL-6 and TNF-α, % stain/mm^2^ by immunohistochemistry, IHC and IL-6, TNF-α and IL-1β, pg/mg protein by ELISA).

## Results

**Table 1:** 

	W-S	W-CNP	SHR-S	SHR-CNP
SBP	118±2	122±3	175±3*	159±5#

LMVI	0,023±0,002	0,024±0,001	0,032±0,001*	0,029±0,001

Myocyte area	349±11	337±10	609±25*	557±35#

% Fibrosis	1,54±0,53	1,78±0,17	6,81±0,40*	3,27±0,67#

IL-6 (IHC)	1,8±1,0	1,3±0,6	19,4±2,7*	2,1±0,7#

TNF-α (IHC)	1,1±0,7	1,0±0,5	15,7±3,4*	1,5±0,9#

*IL-6 (ELISA)*	--	--	100,1±6,2	81,0±9,0

*TNF-α (ELISA)*	--	--	8,27±0,89	4,32±0,18#

*IL-1β (ELISA)*	--	--	135,7±5,8	120,3±2,3#

Values are means ± SEM.

*p<0.01 vs W-S,

# p<0.01 vs SHR-S.

## Conclusions

Characteristic high SBP values in SHR are accompanied by hypertrophy, fibrosis and a higher presence of inflammatory cytokines.

Our results show that chronic treatment with CNP attenuates the expression of proinflammatory markers and the early signs of fibrosis in cardiac tissue of hypertensive rats. These effects, combined with the drop in blood pressure we observed, indicate that CNP could possibly have an important pathophysiological and therapeutical role in preventing or even reversing cardiac fibrosis and inflammation accompanying left ventricular remodelling in arterial hypertension.
